# WDR26 and MTF2 are therapeutic targets in multiple myeloma

**DOI:** 10.1186/s13045-021-01217-9

**Published:** 2021-12-07

**Authors:** Fumou Sun, Yan Cheng, Jesse D. Riordan, Adam Dupuy, Wendy Dubois, Michael Pisano, Jing Dong, Beverly Mock, Fenghuang Zhan, Parameswaran Hari, Siegfried Janz

**Affiliations:** 1 Division of Hematology and Oncology, Department of Medicine, Medical College of Wisconsin, 8701 Watertown Plank Road, MFRC 6033, Milwaukee, WI 53226 USA; 2 Department of Anatomy & Cell Biology, University of Iowa, Iowa City, IA USA; 3 Laboratory of Cancer Biology and Genetics, Center for Cancer Research, National Cancer Institute, Bethesda, MD USA; 4 Interdisciplinary Graduate Program in Immunology, University of Iowa, Iowa City, IA USA; 5 Medical College of Wisconsin Cancer Center, Milwaukee, WI USA; 6 Myeloma Center, Division of Hematology and Oncology, Department of Medicine, and Winthrop P. Rockefeller Cancer Institute, University of Arkansas for Medical Sciences, Little Rock, AR USA

**Keywords:** Forward genetic screen, Moloney murine leukemia virus, Plasma cell neoplasia, Carboxy-terminal to LisH (CTLH) complex, Polycomb repressive complex 2 (PRC2)

## Abstract

Unbiased genetic forward screening using retroviral insertional mutagenesis in a genetically engineered mouse model of human multiple myeloma may further our understanding of the genetic pathways that govern neoplastic plasma cell development. To evaluate this hypothesis, we performed a tumor induction study in MYC-transgenic mice infected as neonates with the Moloney-derived murine leukemia virus, MOL4070LTR. Next-generation DNA sequencing of proviral genomic integration sites yielded rank-ordered candidate tumor progression genes that accelerated plasma cell neoplasia in mice. Rigorous clinical and biological validation of these genes led to the discovery of two novel myeloma genes: *WDR26* (WD repeat-containing protein 26) and *MTF2* (metal response element binding transcription factor 2). WDR26, a core component of the carboxy-terminal to LisH (CTLH) complex, is overexpressed or mutated in solid cancers. MTF2, an ancillary subunit of the polycomb repressive complex 2 (PRC2), is a close functional relative of PHD finger protein 19 (PHF19) which is currently emerging as an important driver of myeloma. These findings underline the utility of genetic forward screens in mice for uncovering novel blood cancer genes and suggest that WDR26-CTLH and MTF2-PRC2 are promising molecular targets for new approaches to myeloma treatment and prevention.

**Supplementary Information:**

The online version contains supplementary material available at 10.1186/s13045-021-01217-9.

## To the Editor,

Multiple myeloma (MM) is a common blood cancer derived from terminally differentiated B-lymphocytes called plasma cells (PCs). Despite recent advancements in treatment options, MM remains incurable in the great majority of cases, with no more than half of patients surviving past 5 years [1]. Reasons for poor outcome include tumor heterogeneity and severe limitations in our knowledge base on genetic pathways that drive neoplastic PC development from an early progenitor stage to frank malignancy. Unbiased genetic forward screening using proviral insertional mutagenesis [2] in a dedicated mouse model of human myeloma may lend itself to attacking this knowledge gap. Here, we employ this approach, for the first time, to discover two candidate genes that may yield new opportunities for molecularly targeted myeloma treatments: *WDR26* (WD repeat-containing protein 26) and *MTF2* (metal response element binding transcription factor 2).

**Fig. 1:**
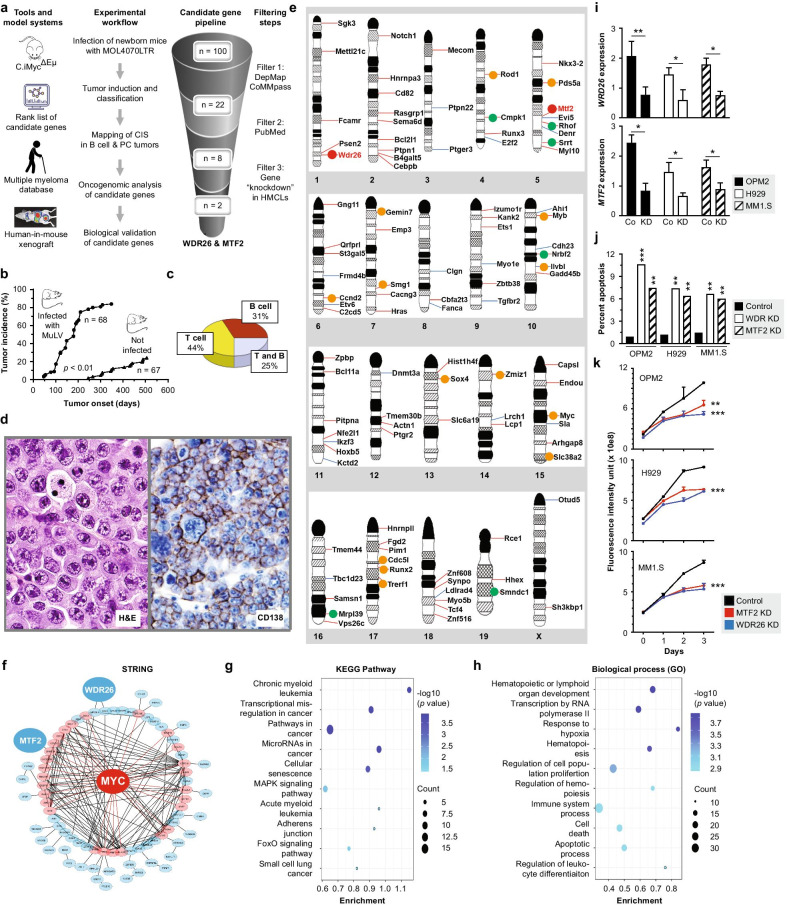
Discovery of *WDR26* and *MTF2* in unbiased genetic forward screen using Myc-transgenic mice. **a** Schema of workflow that led to the nomination of *WDR26* and *MTF2* as candidate myeloma genes. Filters used to pare down the list of 100 input genes to 2 candidate genes are indicated on the right. **b** Accelerated tumor development in iMyc^ΔEµ^ gene-insertion mice treated with MOL4070LTR (mean tumor onset 178 ± 94 days; range 46–348 days) compared to mice not infected with virus (mean tumor onset 384 ± 86 days; range 245–505 days). Virus was injected IP (5 × 10^4^ colony forming units/10 µL) using a 30-gauge needle. **c** Tumor pattern in virus-infected mice from **b**. **d** Tissue section of plasmacytoma (left) and B lymphoma exhibiting plasmablastic differentiation (right) stained according to hematoxylin and eosin (H&E) and immunostained using antibody to CD138, respectively. **e** Ideogrammatic representation of mouse autosomes plus chromosome X indicating the genomic location of the top 100 candidate B cell and plasma cell tumor genes detected. Genes that passed Filters 1, 2 and 3 in **a** are labeled using orange, green and red dots, respectively. Thin red or blue lines denote whether cRIS mapping predicts increased or decreased gene expression due to proviral insertion. **f** Network of MYC-interacting proteins visualized by STRING (Search Tool for the Retrival of Interacting Genes). Proteins (n = 27) that interact with MYC directly or indirectly are depicted in red or blue, respectively. The minimum required interaction score was 0.5. Red and black lines within the network circle denote direct and indirect interactions with MYC, respectively. The symbols for WDR26 and MTF2 are enlarged for enhanced visibility. Network visualization relied on Cytoscape 3.8.2. **g** KEGG (Kyoto Encyclopedia of Genes and Genomes) pathway analysis of the top 100 candidate genes. Enrichment scores are denoted by ovals which indicate both the number of pathway genes involved (count) and level of statistical significance (blue saturation). **h** GO (Gene Ontology) term enrichment analysis of biological processes using the top 100 candidate genes from **a** as input. **i** Magnitude of RNAi-dependent knockdown (KD) of *WDR26* and *MTF2* expression in three different HMCLs relative to HMCLs transfected with scrambled message (control). **j** Programmed cell death in HMCLs exhibiting low *WDR26* or *MTF2* expression (KD) compared to HMCLs cells containing normal message levels (control). Gene knockdown relied on Sigma MISSION® Endoribonuclease-prepared siRNAs (esiRNAs, 50 ng), a heterogeneous mix of siRNAs that target the same message. **k** Growth inhibition (PrestoBlue™) of HMCLs harboring low levels of *WDR26* or *MTF2* message. Genes were knocked down using Mission EHU esiRNAs 150,671 (WDR26) and 042,951 (MTF2)

Our experimental strategy for detecting presumptive therapeutic targets in MM is depicted in Fig. 1a. The first step was a tumor induction study in iMyc^ΔEµ^ mice, a gene-insertion model of the chromosomal T(12;15) translocation that results in deregulated expression of *Myc* in B-lineage cells [3]. Because T(12;15) is a tumor-initiating event in mouse plasmacytoma [4] and upregulation of *MYC* is a well-established mechanism of tumor progression in human myeloma [5], the iMyc^ΔEµ^ transgene served as an ideal “sensitizer” for skewing the oncogenic potency of the murine leukemia virus (MuLV), MOL4070LTR, to plasmablasts and PCs. MOL4070LTR is a modified Moloney-MuLV that contains the LTR U3 enhancer region from the amphotropic MuLV, 4070A [6]. Infection of newborn iMyc^ΔEµ^ mice with MOL4070LTR resulted in accelerated tumor development (Fig. 1b): 51 of 68 (75%) virus-treated mice developed tumors by 210 days of age, whereas less than a quarter of untreated mice demonstrated malignant growth by 505 days. Histopathological tumor classification relied on immunostaining for T cell (CD3), B cell (Pax5, B220) and PC (CD138) markers to assign tumor-bearing mice with virus-accelerated neoplasms to the B-lineage (31%) or T-lineage (44%). A quarter of mice (25%) contained both B- and T-cell tumors (Fig. 1c). From all mice carrying B-lineage tumors (n = 21), eight individual tumor samples (spleen plus peripheral and deep lymph nodes) were collected on average. Most tumors were categorized as plasmacytoma (Fig. 1d, left) or plasmablastic lymphoma (Fig. 1d, right) in accordance with the Bethesda proposal of lymphoid tumors in mice [7]. A total of 168 tumor specimens were analyzed for common retroviral insertion sites (CIS) as depicted in Additional file 1: Fig. S1. From nearly half a million mapped sequence reads, approximately 45 thousand proviral integration events were extracted. To unequivocally identify CIS, we used a biocomputational algorithm based on Monte Carlo statistics that considered both the number of independent integration sites in a given DNA window and the distance between the sites. We defined a CIS as the minimum genomic region in which 5 to 7 unique insertions were found to be significant at *p* < 0.05, provided that no more than two insertions were derived from the same tumor. CIS windows ranged from 10 to 40 kb, corresponding to the size of the transcriptional unit of the average mouse gene (~ 30 kb). A total of 171 CIS-tagged candidate genes were identified and rank ordered according to proviral insertion frequency. The top 100 genes are shown in Fig. 1e. Included are many genes one might have expected in a forward genetic screen of neoplastic PC development; e.g., *Ccnd2* on Chr 6, *Hras* on Chr 7 and *Myc* on Chr 15.

Bioinformatics analysis of the top 100 genes using STRING (string-db.org) demonstrated their tight association with the oncogenic MYC network (Fig. 1f). KEGG analysis (www.kegg.jp) revealed significant enrichment in cancer-relevant pathways including blood cancers such as AML and CML (Fig. 1g). GO analysis of biological processes (geneontology.org) demonstrated strong enrichment in pathways of hematopoiesis, hematopoietic or lymphoid organ development, and regulation of leukocyte differentiation (Fig. 1h). These results underscored the relevance of the top 100 genes for MM and encouraged us to narrow them down to the most promising candidates. This process began with two steps denoted “Filter 1” in Fig. 1a, top right. The first step asked the question whether *upregulation* of the human orthologs of the top 100 mouse genes predicted to be *upregulated* by proviral insertion might be associated with inferior survival in human myeloma. We chose the MMRF CoMMpass study to test for associations of gene expression and survival because this study evaluates outcomes in over one thousand patients in a publicly accessible fashion (https://research.themmrf.org). The second filtering step relied on the DepMap data explorer tool (depmap.org/portal), which provides CRISPR and RNAi dependency scores that indicate whether a gene of interest is important and functionally non-redundant in myeloma in vitro. Twenty-two of the top 100 mouse genes (labeled with colored dots in Fig. 1e) passed Filter 1. Next, we performed a rigorous PubMed analysis of the 22 genes for published evidence on their involvement in MM and related diseases (“Filter 2” in Fig. 1a). Only eight genes (green and red in Fig. 1e) promised novelty for myeloma. These candidates proceeded to “Filter 3” in Fig. 1a, which assessed whether shRNA-mediated knockdown (KD) of gene expression inhibited myeloma in cell culture. Three HMCLs were transfected with eight different Mission EHU esiRNAs, and gene KD was verified by qPCR in 8 of 8 cases (not shown). However, significant (*p* < 0.01) and consistent inhibition (in 3 of 3 cell lines) was only seen in two cases: WDR26 and MTF2 (Fig. 1i–k).

**Fig. 2:**
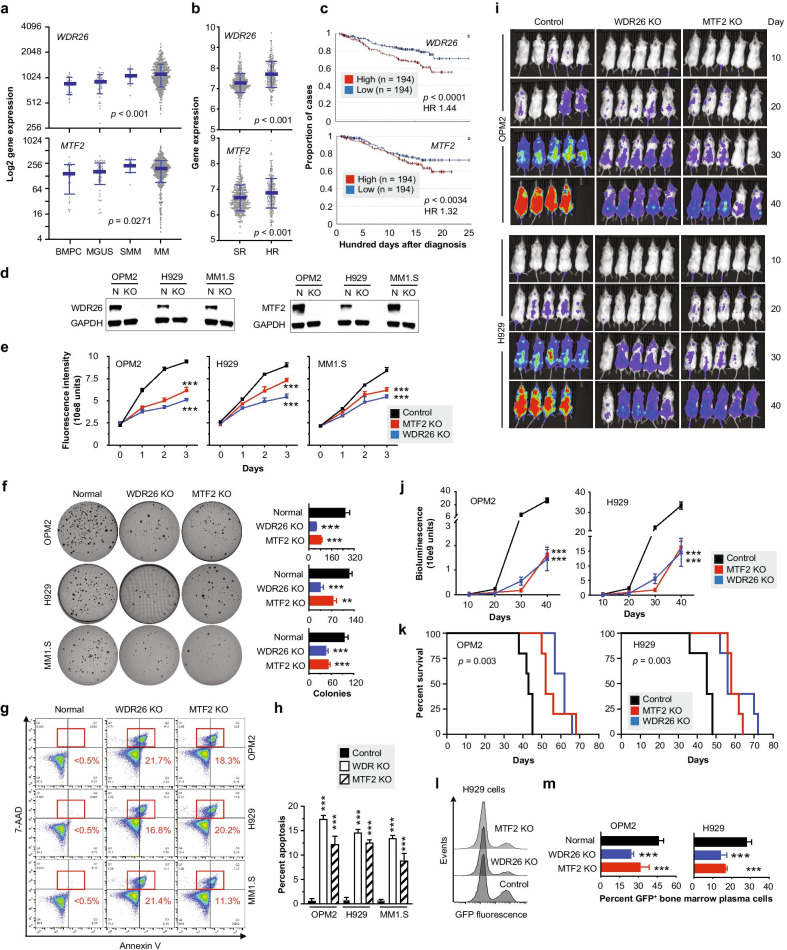
Clinical and biological validation of WDR26 and MTF2 in myeloma. **a** *WDR26* (top) and *MTF2* (bottom) expression levels (GSE 2658 and 5900 datasets) in bone marrow plasma cells from healthy individuals (BMPC, n = 22) or patients with monoclonal gammopathy of undetermined significance (MGUS, n = 44), smoldering myeloma (SMM, n = 12) or frank myeloma (MM, n = 559). MM data are from GSE2658, all others from GSE5900. **b** Comparison of mean mRNA levels of *WDR26* (top) or *MTF2* (bottom) in patients with standard-risk myeloma (SR, n = 690) or high-risk myeloma (HR, n = 287), using data from the Multiple Myeloma DREAM Challenge study. **c** Overall survival (OS) of patients with myeloma in the MMRF CoMMpass study stratified according to *WDR26* (top) or *MTF2* (bottom) message levels in malignant plasma cells. The top quartile (n = 194, red) and bottom quartile (n = 194, blue) are compared. HR, hazard ratio. **d** Western analysis of WDR26 (left) or MTF2 (right) in normal (N) or gene-targeted (KO) myeloma cell lines, OPM2, H929 and MM1.S. KO protocols including gRNA sequences are available upon request. **e** Growth of HMCLs in bulk suspension culture. Cells deficient in WDR26 (blue) or MTF2 (red) are compared to parental cells (black) used as control. **f** Clonogenic growth of OPM2 (top), H929 (center) and MM1.S cells (bottom) lacking WDR26 (blue) or MTF2 (red) or containing the proteins (black). Representative images of soft-agar plates are shown to the left. The bar diagram to the right displays mean colony numbers ± SD based on three independent experiments. **g** Representative flow cytometric scatter plots of apoptotic death (red, labeled rectangles) of WDR26 or MTF2 deficient HMCLs compared to normal cells. **h** Mean values of apoptosis based on three independent measurements. Standard deviations are indicated by short vertical lines (***p < 0.001). **i** Bioluminescence images of NSG mice on days 10, 20, 30 and 40 following challenge with OPM2 cells (upper panel) or H929 cells (lower panel) deficient of WDR26 (center column) or MTF2 (right column). Parental cells proficient of these proteins served as control (left column). **j** Quantitative analysis of bioluminescence signal strength in mice from **h**. **k** Kaplan–Meier survival curves of mice depicted in **h**. Statistical comparison relied on log rank analysis. **l** Flow cytometry histogram distinguishing GFP-expressing tumor cells in the bone marrow of xenotransplanted mice from **h** (smaller peaks, right) from bone marrow cells not expressing GFP (larger peaks, left). **m** Abundance of GFP-expressing tumor cells in the bone marrow of mice from **h**

To validate WDR26 and MTF2 in greater depth, we gathered additional clinical and biological data (Fig. 2). Clinical results in support of the contention that *WDR26* and *MTF2* are important in MM include the circumstance that gene expression was upregulated in smoldering and frank myeloma (Fig. 2a), that message levels in the DREAM Challenge study [8] were elevated in high-risk compared to standard-risk myeloma (Fig. 2b) and that high amounts of mRNA in myeloma cells of CoMMpass patients predicted inferior overall survival (Fig. 2c). To strengthen the biological evidence on the impact of WDR26 and MTF2 in MM, we complemented the KD data shown in Fig. 1i–k with loss-of-function studies using CRISPR-Cas9 engineered gene knockouts (KO) in myeloma cells (Fig. 2d). WDR26 or MTF2 deficiency compromised the growth of myeloma in both bulk suspension (Fig. 2e) and clonogenic soft-agar culture (Fig. 2f). KO led to a significant increase in apoptotic cell death measured with the help of annexin V immunoreactivity (Fig. 2g, h). In vivo studies using HMCL-in-mouse xenografts added further confidence to these results: WDR26 or MTF2 deficient tumors grew more slowly than their normal counterparts (Fig. 2i, j) and thus permitted longer survival of host mice (Fig. 2k). Employment of GFP as reporter of malignant growth produced similar results; e.g., the abundance of tumor cells in the bone marrow of mice harboring WDR26 or MTF2 deficient myeloma was cut in half compared to controls (Fig. 2l, m).

In conclusion, this study used a sensitized forward genetic screen in laboratory mice to nominate *WDR26* and *MTF2* as candidate myeloma genes. WDR26 is a component of the CTLH complex that is mutated or upregulated in many solid cancers [9]. WDR26 has not been implicated in blood cancers, yet its significance as therapeutic target in carcinomas has been recognized [10]. MTF2, an accessory unit of the PRC2 complex involved in gene repression and growth promotion of various cancers [11], is a validated molecular target in AML [12]. MTF2 is new in myeloma, but PHF19, another accessory unit of PRC2, has emerged as a major player in MM [8, 13–15]. Both MTF2 and PHF19 are preferentially overexpressed in high-risk myeloma. Additional research is warranted to elucidate the oncogenic networks of WDR26 and MTF2 in myeloma because this may point to new avenues for molecularly targeted treatments and preventions.

## Supplementary Information

**Additional file 1**.** Fig. S1**. Identification of proviral integration sites and candidate driver genes. Genomic DNA was extracted from malignant tissues harvested from MOL4070LTR-infected mice. Approximately 1 μg of genomic DNA was then digested using either MseI or NlaIII. Next, 200 ng of digested DNA was ligated to double-stranded adaptors (NlaIII linker: 5’-GTA ATA CGA CTC ACT ATA GGG CTC CGC TTA AGG GAC CAT G-3’ and 5’-Phos-GTC CCT TAA GCG GAG-C3spacer-3’, MseI linker: 5’-GTA ATA CGA CTC ACT ATA GGG CTC CGC TTA AGG GAC-3’ and 5’- Phos-TAG TCC CTT AAG CGG AG-C3spacer-3’). Following adaptor ligation, DNA was digested with EcoRV to eliminate the internal proviral fragment (indicated by red cross). EcoRV-digested DNA was then amplified (primary PCR) using primers annealing to the adaptor (5’-GTA ATA CGA CTC ACT ATA GGG CTC CG-3’) and the proviral LTR (5’-GCT AGC TTG CCA AAC CTA CAG GTG G-3’). PCR products were diluted 1:50 in sterile water. Two microliters of diluted PCR product was re-amplified (secondary PCR) using nested primers annealing to the adaptor (5’-AGG GCT CCG CTT AAG GGA C-3’) and proviral LTR (5’-CCA AAC CTA CAG GTG GGG TCT TTC-3’). Amplicons from the second round of PCR were purified to remove unincorporated primers and nucleotides and directly sequenced on an Illumina platform. Raw sequences were trimmed to remove adaptors and viral sequences and mapped to the mouse reference genome. Candidate driver genes were identified using Monte Carlo simulation as previously described (PMID: 21931803).

## Data Availability

Detailed information on materials and methods used, including KD and KO primer sequences, are available from the corresponding author upon request.

## Abbreviations

AAD: 7-Aminoactinomycin D
AML: Acute myeloid leukemia
CML: Chronic myeloid leukemia
CIS: Common retroviral insertion site
CTLH: Carboxy-terminal to LisH
GFP: Green fluorescent protein
HMCL(s): Human myeloma cell line(s)
LTR: Long terminal repeat
KD: Knockdown
KO: Knockout
LTR: Long terminal repeat
MM: Multiple myeloma
MMRF: Multiple Myeloma Research Foundation
MOL4070LTR: Moloney derived MuLV
MTF2: Metal response element binding transcription factor 2
MuLV: Murine leukemia virus
NGS: Next-generation sequencing
PC(s): Plasma cell(s)
PRC2: Polycomb repressive complex 2
WDR26: WD repeat-containing protein 26
