# Supplementary material for: WDR26 and MTF2 are therapeutic targets in multiple myeloma
Source: J Hematol Oncol. 2021 Dec 7;14:203. doi: 10.1186/s13045-021-01217-9 (PMC8650373; doi:10.1186/s13045-021-01217-9)
Supplement: Supplementary file 1 — Additional file 1. Fig. S1. Identification of proviral integration sites and candidate driver genes. Genomic DNA was extracted from malignant tissues harvested from MOL4070LTR-infected mice. Approximately 1 μg of genomic DNA was then digested using either MseI or NlaIII. Next, 200 ng of digested DNA was ligated to double-stranded adaptors (NlaIII linker: 5’-GTA ATA CGA CTC ACT ATA GGG CTC CGC TTA AGG GAC CAT G-3’ and 5’-Phos-GTC CCT TAA GCG GAG-C3spacer-3’, MseI linker: 5’-GTA ATA CGA CTC ACT ATA GGG CTC CGC TTA AGG GAC-3’ and 5’- Phos-TAG TCC CTT AAG CGG AG-C3spacer-3’). Following adaptor ligation, DNA was digested with EcoRV to eliminate the internal proviral fragment (indicated by red cross). EcoRV-digested DNA was then amplified (primary PCR) using primers annealing to the adaptor (5’-GTA ATA CGA CTC ACT ATA GGG CTC CG-3’) and the proviral LTR (5’-GCT AGC TTG CCA AAC CTA CAG GTG G-3’). PCR products were diluted 1:50 in sterile water. Two microliters of diluted PCR product was re-amplified (secondary PCR) using nested primers annealing to the adaptor (5’-AGG GCT CCG CTT AAG GGA C-3’) and proviral LTR (5’-CCA AAC CTA CAG GTG GGG TCT TTC-3’). Amplicons from the second round of PCR were purified to remove unincorporated primers and nucleotides and directly sequenced on an Illumina platform. Raw sequences were trimmed to remove adaptors and viral sequences and mapped to the mouse reference genome. Candidate driver genes were identified using Monte Carlo simulation as previously described (PMID: 21931803). [file 13045_2021_1217_MOESM1_ESM.pdf]

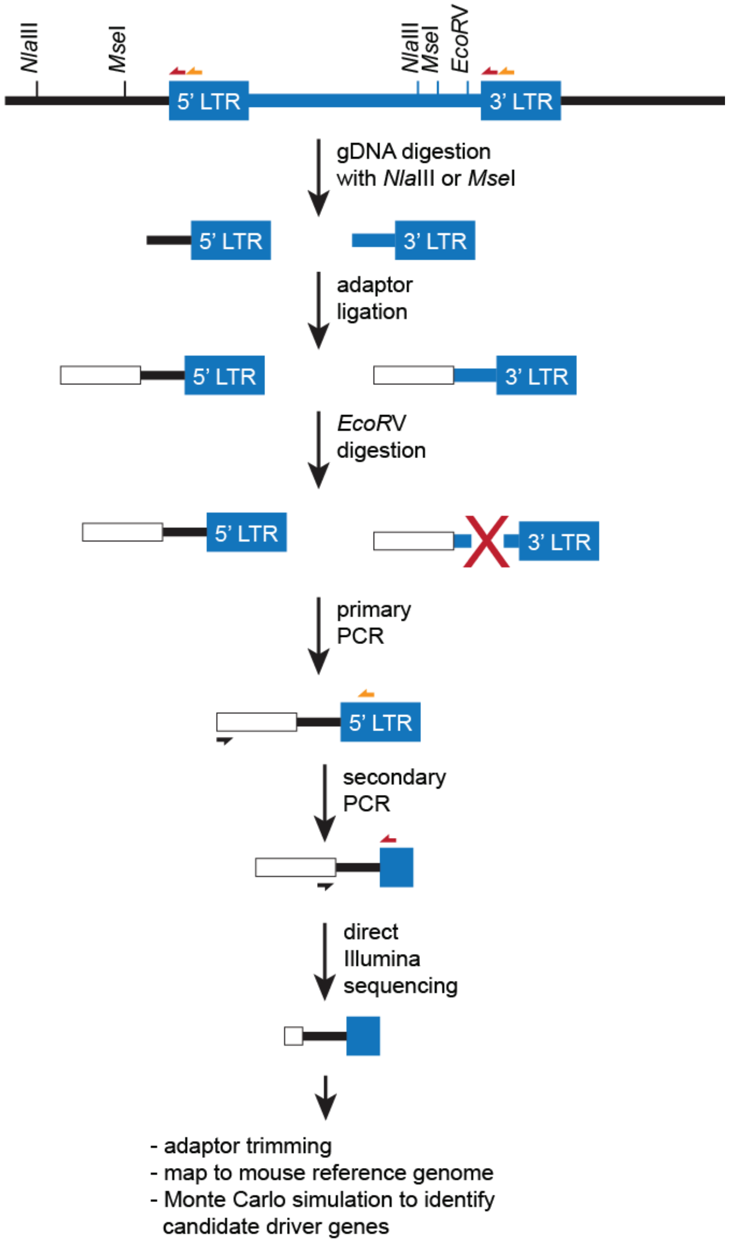

Genomic DNA was extracted from malignant tissues harvested from MOL4070LTR-infected mice. Approximately 1 µg of genomic DNA was digested using either MseI or NlaIII. Next, 200 ng of digested DNA was ligated to double stranded adaptors (NlaIII linker: 5'-GTAATAC-GACTCACTATAGGGCTCCGCTTAAGGGAC-CATG-3' and 5'-Phos-GTCCCTTAAGCG-GAG-C3spacer-3', MseI linker: 5'-GTAATAC-GACTCACTATAGGGCTCCGCTTAAGGGAC-3' and 5'-Phos-TAGTCCCTTAAGCG-GAG-C3spacer-3'). Following adaptor ligation, DNA was digested with EcoRV to eliminate the internal proviral fragment (indicated by red cross). EcoRV-digested DNA was then amplified (primary PCR) using primers annealing to the adaptor (5'-GTAATACGACTCACTATAGG-GCTCCG-3') and the proviral LTR (5'-GCTAGCTTGCCAAACCTACAGGTGG-3'). PCR products were diluted 1:50 in sterile water. Two microliters of diluted PCR product was re-amplified (secondary PCR) using nested primers annealing to the adaptor (5'-AGG-GCTCCGCTTAAGGGAC-3') and proviral LTR (5'-CCAAACCTACAGGTGGGGTCTTTC-3'). Amplicons from the second round of PCR were purified to remove unincorporated primers and nucleotides and directly sequenced on an Illumina platform. Raw sequences were trimmed to remove adaptors and viral sequences and mapped to the mouse reference genome. Candidate driver genes were identified using Monte Carlo simulation as previously described (PMID: 21931803).

Supplemental Figure 1: Sun et al.
